# What the Local Centres Are Doing

**Published:** 1921-01-29

**Authors:** 


### THE COLLEGE OF NURSING

*(Limited by Guarantee).*

### WHAT THE LOCAL CENTRES ARE DOING.

### BIRMINGHAM THREE COUNTIES CENTRE.

(Hon. Secretary, Mrs. M. GLEGG, 84 Hagley Road, Birmingham.)

SATURDAY, JANUARY 29, 3.30 P.M.—Business meeting in the Lecture Theatre of the General Hospital, Birmingham (by kind permission of the Governors), to receive the Hon. Secretary's and Hon. Treasurer's reports. Subsequently an address will be given by Miss Musson, R.R.C., Local Representative, and at 4 o'clock tea will be served in the Board Room. Tickets, 1s.

### GLASGOW CENTRE.

(Hon. Secretary, Miss E. MOSELEY, Western District Hospital, Oakbank.)

AT a meeting held on SATURDAY, JANUARY 22, in the Glasgow Nurses' Club, 10 Claremont Terrace, W., by kind permission of Miss Roy Reid, Miss Craig Roberton (member of Glasgow Parish Council) gave an interesting lecture on "Citizenship." The great importance of women using their power to vote upon all occasions was emphasised, and Miss Roberton compared the status and work of women in different parts of the British Dominions which she has lately visited. Miss Melrose, R.R.C., presided, and at the conclusion a hearty vote of thanks was accorded to Miss Roberton for her able and interesting lecture.

### LIVERPOOL CENTRE.

(Hon. Secretary, Miss ASPINALL, Stanley Hospital.)

**Police Work as a Profession for Women.**

MISS M. H. COWLIN, Director of the Liverpool Training School for Women Police, gave an address on this subject on JANUARY 18. Miss Cowlin explained that women police are needed not as substitutes for men, but to do the work amongst women and children that women alone can do. This includes patrol duty in streets and open spaces, where they need the power of arrest to deal with drunken and immoral women. She urged that in Police Courts women police should have complete charge of women and children either as offenders or as offended against, and should undertake all inquiries in cases of assault on women and children. Much of the work undertaken by policewomen would prove to be preventive work, but preventive work is the true foundation of all good police work, and their special ability for this aspect of the work should encourage the police authorities to appoint women police without delay.

The following resolution was unanimously adopted, and the Hon. Secretary asked to forward it to the Home Secretary, the Chief Constable of Liverpool, and the Chairman of the Watch Committee:

"That the members of the Liverpool Branch of the College of Nursing earnestly request the Home Secretary to urge the Chief Constable and the Watch Committee of Liverpool to appoint without delay an adequate number of trained women police for patrol work in the streets and open spaces, and for work amongst women and children in Police Courts, in accordance with the recommendations of the report of the Committee on the Employment of Women on Police Duties."

### LONDON CENTRE.

(Secretary, Miss BOMPAS, 7 Henrietta Street, W. 1.)

MONDAY, FEBRUARY 7, 8 P.M.—At the College of Ambulance, 56 Queen Anne Street, W. 1, lecture on "The Relation between Mental Nursing and Hospital Nursing," by Sir Robert Armstrong-Jones, M.D.

### YORKSHIRE CENTRE, LEEDS.

(Hon. Secretary, Miss LINDALL, Women's and Children's Hospital.)

A MOST enjoyable dance was held on FRIDAY, JANUARY 21, at the Hospital for Women and Children, Leeds, when sixty members were present. A card-room was arranged for those not wishing to dance, but the dance-room claimed the greater number.

During the evening a letter was read from the Hon. Secretary of the London Centre dealing with nominations for the Council elections. The letter suggested representation of provincial Poor-law, or special hospital, or the working nurse. Seeing that the members were assembled for physical, not mental, exercise, it is not surprising that no decision was arrived at, but a meeting is to be held in February to nominate candidates.

